# Training for health services and systems research in Sub-Saharan Africa - a case study at four East and Southern African Universities

**DOI:** 10.1186/1478-4491-11-68

**Published:** 2013-12-23

**Authors:** David Guwatudde, Freddie Bwanga, Lilian Dudley, Lumbwe Chola, Germana Henry Leyna, Elia John Mmbaga, Newton Kumwenda, Myroslava Protsiv, Salla Atkins, Merrick Zwarenstein, Celestino Obua, James K Tumwine

**Affiliations:** 1 Makerere University College of Health Sciences, Kampala, Uganda; 2 Faculty of Health Sciences, University of Stellenbosch, Cape Town, South Africa; 3 Department of Epidemiology and Biostatistics, Muhimbili University of Health and Allied Sciences, Dar-es-Salam, Tanzania; 4 College of Medicine, University of Malawi, Blantyre, Malawi; 5 Department of Public Health Sciences, Karolinska Institutet, 171 77, Stockholm, Sweden; 6 Institute of Health Policy, Management and Evaluation, University of Toronto, Toronto, ON, Canada; 7 School of Public Health, Makerere University College of Health Sciences, PO Box 7072, Kampala, Uganda; 8 Department of Medical Microbiology, School of Biomedical Sciences, Makerere University College of Health Sciences, Kampala, Uganda; 9 Department of Pharmacology and Therapeutics, School of Biomedical Sciences, Makerere University College of Health Sciences, Kampala, Uganda; 10 Department of Pediatrics and Child Health, School of Medicine, Makerere University College of Health Sciences, Kampala, Uganda

**Keywords:** Health services and systems research, training, Sub-Saharan Africa

## Abstract

**Background:**

The need to develop capacity for health services and systems research (HSSR) in low and middle income countries has been highlighted in a number of international forums. However, little is known about the level of HSSR training in Sub-Saharan Africa (SSA). We conducted an assessment at four major East and Southern African universities to describe: a) the numbers of HSSR PhD trainees at these institutions, b) existing HSSR curricula and mode of delivery, and c) motivating and challenging factors for PhD training, from the trainees’ experience.

**Methods:**

PhD training program managers completed a pre-designed form about trainees enrolled since 2006. A desk review of existing health curricula was also conducted to identify HSSR modules being offered; and PhD trainees completed a self-administered questionnaire on motivating and challenging factors they may have experienced during their PhD training.

**Results:**

Of the 640 PhD trainees enrolled in the health sciences since 2006, only 24 (3.8%) were in an HSSR field. None of the universities had a PhD training program focusing on HSSR. The 24 HSSR PhD trainees had trained in partnership with a university outside Africa. Top motivating factors for PhD training were: commitment of supervisors (67%), availability of scholarships (63%), and training attached to a research grant (25%). Top challenging factors were: procurement delays (44%), family commitments (38%), and poor Internet connection (35%).

**Conclusion:**

The number of HSSR PhD trainees is at the moment too small to enable a rapid accumulation of the required critical mass of locally trained HSSR professionals to drive the much needed health systems strengthening and innovations in this region. Curricula for advanced HSSR training are absent, exposing a serious training gap for HSSR in this region.

## Introduction

Health systems and services research (HSSR)has been defined as ‘a field of study that examines the organization, financing, and delivery of public health services within communities and the impact of those services’ [1]. HSSR capacity is the set of skills and competencies permitting the conduct of research to strengthen health services and systems and consists of six building blocks including: service delivery, information and evidence, medical products and technologies, health work force, health financing and leadership, and governance [2]. Building a robust health system that is responsive and adequately addresses the health of a country therefore requires trained professionals that have the skills to gather, analyze and generate necessary evidence to inform appropriate health policy. It would include the ability to prioritize health challenges, select the best approaches to solve a problem, adapt them to local contexts and test these in real-world conditions, and deciding upon wider implementation decisions.

Over the past decade, numerous calls have been made to strengthen HSSR capacity in low and middle income countries (LMICs) as one of the ways to improve health care delivery in these settings. The calls have been made through scientific publications [3-7] and a number of international events and reports including, but not limited to: the Mexico Ministerial Summit of 2004 [8], the Bamako Ministerial Summit of 2008 [9], and the First and Second Global Symposia on Health Systems Research of 2010 and 2012 respectively [10,11]. This is in recognition of the poorly performing health systems in many LMICs, which impede improvement of health care delivery. Despite these initiatives, health outcome indicators in most of Sub-Saharan Africa (SSA) suggest little progress towards improvement of health systems responsiveness; and most of the SSA countries are lagging behind in achieving the health Millennium Development Goals (MDGs) [12,13].

It is widely recognized that if health care delivery in SSA is to improve, health systems strengthening is a key factor towards this effort [13-15]. Progress is dependent upon having a sufficient complement of scientists with the professional expertise to undertake relevant, timely and reliable research; and the ability to support policy makers in translating research into policy and practice [8,16,17]. Presently, however, there are not enough HSSR professionals to do this in SSA. Little attention has been paid to building adequate HSSR capacity through training of HSSR professionals within SSA. Although many institutions in developed economies can provide this kind of training, the required critical mass of HSSR professionals in SSA can be achieved faster and sustained if the capacity to train these professionals is developed within SSA. A literature search on the current level of HSSR related training within SSA institutions did not yield any published investigation in this area.

The African Regional Capacity Development for Health Services and Systems Research (ARCADE-HSSR) project aims at increasing the number of HSSR professionals through development of web-based and freely available modules and blended courses delivered in a collaborative way across several universities; which it is hoped will increase access to specialized training [18]. The ARCADE-HSSR project is being implemented in four SSA universities, namely, Makerere University (Uganda), Stellenbosch University (South Africa), Muhimbili University of Health and Allied Sciences (Tanzania), and the University of Malawi (Malawi), with support from northern institutions and the European Union. To provide baseline information about HSSR related training in SSA institutions, the ARCADE-HSSR project consortium partners conducted an assessment to: a) describe the numbers and proportion of HSSR graduates among all health science PhD trainees, b) identify motivating and challenging factors towards PhD training from the trainees’ point of view, c) identify existing HSSR related training modules at these institutions and mode of delivery, and, d) identify areas for PhD training that require capacity development. Our study focused on PhD level training because we view this level of training as the foundation for developing a strong HSSR capacity in a country.

## Materials and methods

### Setting and participants

The assessment was conducted between May and October 2011, at the four East and Southern African universities that are part of the ARCADE-HSSR consortium. Only health science departments within these universities were involved in the assessment. Respondents included current and recently graduated PhD trainees, academic program registrars, and PhD training program managers.

### Data collection

PhD training program managers in the health science departments were contacted and requested to complete a pre-designed form that sought information on the number of current PhD trainees and those that graduated between 2006 and 2011. The information collected included: trainees’ age, sex, field of study, academic department, year of enrollment, and year of graduation for those that had already graduated. No identifying information such as names and registration numbers were collected. In-depth interviews were also conducted with PhD training program managers to assess available infrastructure and information and communication technology (ICT) facilities at these institutions that can facilitate advanced training in the health sciences, including computer facilities, Internet connectivity and video-conferencing equipment for students.

To identify motivating and challenging factors towards PhD training from the trainees’ experience, current PhD students that had been on the training program for at least two years, and those that had graduated no longer than two years prior to 2011 and could be traced, were sent a self-administered questionnaire via Email or direct contact, which they were requested to complete and return. The self-administered questionnaire sought information on demographics, year of enrollment and graduation, field of study, and type of training arrangement, that is, whether the student studied full-time at the home university, or was part of a ‘sandwich’ arrangement (with a fraction of the training time spent at the home university, and the remaining fraction of time spent at an away-from-home university). The questionnaire also sought information on their experiences on: a) motivating and challenging factors to enroll into the PhD training program, b) motivating and challenging factors faced during the PhD training, and, c) any gender and social issues that may have affected their enrollment or that they had faced during the PhD training.

Finally, a desk review of existing health curricula at these institutions was conducted to identify the various HSSR related modules being offered for masters and PhD training; and the mode of delivery for each module, that is, whether the module was offered in face-to-face mode or in distance-learning mode, and/or if it was freely available on the web.

### Data analysis

Descriptive statistics were used to describe the characteristics of the PhD trainees. To describe the various fields of study of the PhD trainees, the fields of study were classified into nine categories including: a) health systems, b) health service delivery/research, c) health policy/planning, d) health economics/financing, e) health human resource, f) governance in health, g) biomedical sciences, h) clinical sciences, and i) others.

During the review of health science curricula, a number of HSSR related modules being offered were identified, and although some of these had varying names at the different universities, the content for some was generally similar. Modules with generally similar content description were classified under the following categories: a) health services research, b) health systems management and research, c) health policy, d) health planning, e) health economics, f) quality of health care, and g) cost-effectiveness analysis. We report the number of modules in these categories, and the percentage being offered via each of the three modes of delivery, that is, face-to-face mode, distance-learning mode, and web-based freely available.

The motivating and challenging factors described by the PhD trainees are reported as percentages of trainees mentioning the given factors.

Minimum, median and maximum values were used to describe the number of computers with Internet connection reported by PhD training program managers as being available for use by PhD trainees in the various health science departments. Similarly, minimum, median and maximum values were used to describe the number of video-conferencing equipment available for use by PhD trainees in the various health science departments.

### Ethics statement

The study was approved by the respective Institutional Review Boards of the four participating universities, that is, from the Higher Degrees Research and Ethics Committee (HDREC) of Makerere University’s School of Medicine; from the Research Ethics Committee of Muhimbili University of Health and Allied Sciences; from the Health Research Ethics Committee (HREC) of the University of Stellenbosch; and from the College of Medicine Research and Ethics Committee (COMREC) of the University of Malawi. Written informed consent was also obtained from each participant completing the self-administered questionnaire.

## Results

### Characteristics of current and recently graduated PhD trainees

**Figure 1:**
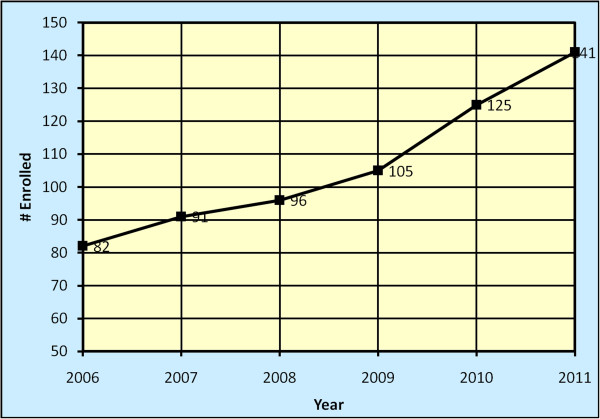
Number of PhD trainees enrolled since 2006.

A total of 640 PhD trainees enrolled in a health science field at the four participating universities between 2006 and 2011. Of these, 359 (56%) were females. The median age at enrollment into the PhD training programs was 37 years with an inter-quartile range of 30 to 44 years. The numbers enrolled progressively increased from 82 in 2006 to 141 in 2011 (see Figure 1), with an annual average over the five-year period of 128.

### Field of study

Of the 640 PhD trainees enrolled between 2006 and 2011, 311 (48%) were reported to be training, or had trained in the fields of biomedical sciences, followed by 254 in the clinical sciences (40%). Only 24 PhD trainees (3.8%) were training or had trained in a HSSR related field, specifically: 23 trainees in health systems (3.6%), and only one in health policy (0.2%). All the 24 students had trained as part of a ‘sandwich’ training program in partnership with a university outside of Africa. No PhD trainee at these institutions had trained in the fields of health service research/delivery, health economics/financing, health human resources or governance in health (Table 1).

**Table 1:** Field of study of current and recently graduated PhD trainees

**Field of PhD study**	**Number of trainees**	**Percentage**
Biomedical sciences	311	48.6%
Clinical sciences	254	39.7%
Health systems	23	3.6%
Health service research/delivery	0	0.0%
Health policy/planning	1	0.2%
Health economics/financing	0	0.0%
Health human resources	0	0.0%
Governance in health	0	0.0%
Others	51	8.0%
Total	640	100.0%

### Modules being offered and mode of delivery

A total of 29 HSSR related modules were identified as currently being offered at the four universities. All were being offered as part of either Masters of Public Health or Masters of Health Services Research. No HSSR related PhD level training was being offered at any of the four universities. Of the 29 HSSR related modules being offered, eight were in health economics, six in health systems management and research, five in health services research, three in health policy, three in health planning, three in quality of health care, and one in cost-effectiveness analysis. All these modules were offered using a face-to-face lecture mode. In addition to being offered as face-to-face mode, three of these, including health policy, health planning and health economics were also offered by distance learning using printed learning materials. Only Makerere University reported to be currently offering a masters degree training program focusing on health services research, with current enrollment of 10 to 15 students per year. None of the participating institutions had web-based freely available HSSR related modules at masters or at PhD level training (Table 2).

**Table 2:** Health services and systems research (HSSR) related modules being offered, and mode of delivery

**Category**	**Number of modules offered in this category**	**Mode of delivery**		
		**In-class**	**Distance learning**	**Web-mounted/online**
Health economics	8	100%	1 (13%)	0%
Health systems management and research	6	100%	0 (0%)	0%
Health services research	5	100%	0 (0%)	0%
Health policy	3	100%	1 (30%)	0%
Health planning	3	100%	1 (30%)	0%
Quality of health care	3	100%	0 (0%)	0%
Cost-effectiveness analysis	1	100%	0 (0%)	0%
Total	29	100%	3 (10%)	0%

### Motivating and challenging factors

The self-administered questionnaire on motivating and challenging factors towards PhD training was sent to a total of 96 PhD trainees that included 78 recently graduated, and 18 current PhD trainees. Of the78 recently graduated PhD trainees, 32 (41%) completed and returned the questionnaire, whereas of the 18 current PhD trainees, 16 (89%) completed and returned the questionnaire; giving an overall total of 48 or overall response rate of 50%. The most commonly cited motivating factors to enroll, or experienced during the PhD training included: high level of supervisors’ commitment (67%), availability of funds to support PhD research (63%), PhD training attached to a research grant (25%), availability of a regular stipend (25%), and availability of downloadable training modules (23%). The leading challenges during PhD training cited were: equipment or materials procurement delays largely due to institutional procedural bureaucracies (44%), lack of funding/scholarships for the training (38%), family commitments (38%), poor Internet connection (35%), conflicting job responsibilities (31%), and inadequate institutional/administrative support (27%). Table 3 summarizes the motivating and challenging factors reported by the PhD trainees.

**Table 3:** Facilitating and challenging factors to enroll, and/or experienced during PhD training

**Facilitating factors**	**n**	**Percentage (out of 48)**
Commitment of supervisors	32	67%
Availability of funds to support PhD research	30	63%
PhD training attached to a research grant	12	25%
Availability of a regular stipend	12	25%
Availability of downloadable training modules	11	23%
‘Sandwich’ type of training program	8	17%
Availability of experts in field of study as supervisor (s)	6	14%
Others	3	6%
**Challenging factors**		
Procurement delays	21	44%
Lack of scholarships	18	38%
Family commitments (especially for females)	18	38%
Poor Internet connection	17	35%
Inadequate institutional/administrative support	13	27%
Lack of reading materials/books	13	27%
Difficulties accessing supervisors	5	10%
Others	6	13%

### ICT facilities for student use

The median number of computers with Internet connection reported in the health departments was ten, with a maximum of sixty. These facilities were open for use to all graduate students (masters and PhD). Moreover, some departments reported having video-conferencing facilities with a median number of zero, and a maximum of three. At all the three institutions, the video-conferencing facilities were reported to serve multi-purposes including video-conferencing with research collaborators and teaching/learning purposes; and were open for use by graduate students and faculty members (Table 4).

**Table 4:** **Information technology facilities in health sciences departments**^**a**^

**Number of departments reporting**		**Number of units available**		
		**Minimum**	**Maximum**	**Median**
Computer units connected to internet				
Makerere University, Uganda	12	0	34	7
Muhimbili University, Tanzania	6	10	21	10.5
University of Malawi, Malawi	11	0	60	5
Overall	29	0	60	10
Video-conferencing facilities				
Makerere University, Uganda	12	0	1	0
Muhimbili University, Tanzania	6	0	1	0
University of Malawi, Malawi	11	0	3	0
Overall	29	0	3	0

^a^The University of Stellenbosch did not participate in this assessment.

## Discussion

Findings from this study show that there has been an average of only five PhD graduates per year in the field of HSSR across the four universities, constituting only 3.8% of all health science PhD trainees. Further, there were no PhD trainees in some of the HSSR fields such as health services research, health economics/financing, governance in health and health human resources. The number of HSSR PhD graduates being produced per year at these universities is very small and is not enough to enable accumulation of the required critical mass of locally trained HSSR professionals to drive the much needed health systems strengthening and innovations in this region. This is a serious training gap in the fields of HSSR that needs to be urgently addressed. There is general agreement that HSSR needs to be driven by local experts who have an intimate understanding of their own health systems and the challenges that they face [4,13,15]. Many SSA countries are lagging behind in achieving the health MDGs, and although there are different reasons for this, the poorly functioning health systems are a major contributing factor [13]. If health care delivery is to be improved and the MDGs achieved in SSA, building local capacity to carryout multidisciplinary research in the context of national health systems must be a priority [5-7,14]. The present lack of capacity in most of the SSA countries to produce human resources with these skills and attributes, is a serious barrier to building effective health systems needed for improved health care delivery and attainment of health MDGs [5,13,19,20].

We also found that HSSR related curricula to facilitate advanced academic training is lacking. All the HSSR related modules being offered at these universities were part of either Masters of Public Health, or Masters of Health Services Research. Absence of HSSR related curricula to support PhD training is most likely one of the reasons why there are few PhD HSSR related training at these universities. Bennett *et al.*[4] have pointed out that health policy and systems research curricula relevant to LMIC is lacking, and where available often occupies a tenuous home within universities and other academic departments [4]. Our findings demonstrate this gap, and indeed of the four universities included in this assessment, only Makerere University presently has a department dedicated to HSSR related training; but even here the department largely focuses on masters training programs. In order for HSSR to develop as a field in these settings, dedicated and supportive homes for HSSR training within universities are required, and there is a need to recognize the inter-disciplinary nature and relevance of HSSR training in these settings as one of the key components in building local capacity for HSSR [4]. We believe that every university should have at least one or two PhD faculty in the HSSR field, and in each country every department in the Ministry of Health and major cities/regions should have a small research unit led by an HSSR PhD graduate, overall requiring at least 50 HSSR PhD graduates in each country. For each of these countries to have 50 PhDs in HSSR therefore, and assuming a career duration of thirty years, we need at least five or seven HSSR PhD graduates per year in each country to service the universities and Ministries of Health alone.

One of the factors pointed out by the PhD trainees that motivated them enrolling into respective PhD training programs was availability of web-based freely available modules that they could access remotely. Trainees that mentioned this were those in a ‘sandwich’ training arrangement under which they were able to freely access web-based modules at a partner northern university. Furthermore, a number of challenges that may limit access to PhD training were also highlighted, for example the need to continue working to earn a living, and family commitment. These findings highlight the need to develop learning modules that can be remotely accessed by those who are not able to leave work, family or other responsibilities, to attend face-to-face training. Innovative ways to increase access to advanced HSSR training in SSA, such as development of web-based freely available modules, like those being developed under the ARCADE-HSSR project [18], need to be devised to provide opportunity to individuals who may not be able to enroll into full-time training programs such as persons with concurrent job and family responsibilities, especially women. Alongside this, is the need to improve ICT infrastructure to facilitate access to such modules, including Internet access. Our assessment revealed that only a few departments in the health sciences at the participating universities had a reasonable number of computers with Internet connection for students. However, in the context of SSA, availability of reliable Internet connection with the necessary bandwidth is a major challenge and prices for Internet connection for the universities in most of these countries still remain high. Previously, in a similar assessment, Gonzalez Block *et al*. [15] found that access to the Internet was absent in 27% of the LMIC institutions assessed [15]. Internet access is an important enabling factor for trainees at tertiary training institutions to access literature and online learning. Indeed if online learning or web-based modules are to be made available, Internet access needs to be improved at SSA training institutions.

### Study limitation

We recognize that our assessment was conducted at only four universities in East and Southern Africa; therefore our findings may have limited generalizability. Nonetheless, the four universities we selected are among the major tertiary training institutions in health sciences in this region. Therefore the numbers and proportion of HSSR trainees may not be very different from other universities within the region. We therefore believe that the gaps in training exposed by our study reasonably reflect the situation in the region.

Secondly, although we found low numbers of HSSR trained PhDs from these universities, HSSR research capacity in this region is enhanced by graduates who are being trained outside of Africa, but return to work in their home countries. Our study did not assess this component; therefore the number of HSSR graduates working in the region might be higher. The desire, however, is to build enough local HSSR training capacity within the region. The ARCADE-HSSR project is trying to address this gap.

Finally, we recognize that our study did not evaluate the number of trainers (faculty) and their capacity to train in the area of HSSR. This investigation was also only conducted at institutions of learning targeting formal education in HSSR, and therefore did not assess other capacities for HSSR training including: perceptions of HSSR capacity and challenges faced by other stakeholders such as donor agencies and non-governmental organizations, networking, sharing information, and transferring knowledge. Thus, our assessment is deficient of other components of capacity for HSSR training. These could be areas for further investigation in the future.

## Conclusions

The current level of HSSR PhD training at SSA institutions is too low to enable the rapid creation of a critical mass of locally trained HSSR professionals to drive health system strengthening and innovations. Further, curricula for advanced HSSR training are absent, exposing a serious training gap for HSSR in this region. HSSR related curricula/modules for advanced HSSR training need to be developed at SSA institutions, in order to enhance the number of experts in this field.

## Abbreviations

ARCADE: African regional capacity development; HSSR: Health services and systems research; ICT: Information and communication technology; LMIC: Low and middle income countries; MDG: Millennium development goals; PhD: Doctor of philosophy; SSA: Sub-Saharan Africa; WHO: World Health Organization.

## Competing interests

The authors declare that there is no conflict of interest.

## Authors’ contribution

DG drafted the manuscript all authors provided comment, read and approved the final version of the manuscript. Study conceptualization: JKT, CO, DG, MZ. Data collection: FB, DG, JKT, LD, LC, GHL, ELM, NK, MP, SA. Data management and analysis: DG and FB.
